# A reduced SNP panel to trace gene flow across southern European wolf populations and detect hybridization with other *Canis* taxa

**DOI:** 10.1038/s41598-022-08132-0

**Published:** 2022-03-09

**Authors:** Astrid Vik Stronen, Federica Mattucci, Elena Fabbri, Marco Galaverni, Berardino Cocchiararo, Carsten Nowak, Raquel Godinho, Aritz Ruiz-González, Josip Kusak, Tomaž Skrbinšek, Ettore Randi, Albena Vlasseva, Nadia Mucci, Romolo Caniglia

**Affiliations:** 1 Department of Biology, Biotechnical Faculty, University of Ljubljana, Večna pot 111, 1000 Ljubljana, Slovenia; 2 Department of Biotechnology and Life Sciences, Insubria University, via J. H. Dunant 3, 21100 Varese, Italy; 3 Department of Chemistry and Bioscience, Aalborg University, Fredrik Bajers Vej 7H, 9220 Aalborg, Denmark; 4 Unit for Conservation Genetics (BIO-CGE), Italian Institute for Environmental Protection and Research (ISPRA), Via Cà Fornacetta 9, 40064 Ozzano dell’ Emilia, Bologna, Italy; 5 Science Unit, WWF Italia, Via Po 25c, 00198 Rome, Italy; 6 Wildlife Genetics Center, Senckenberg Research Institute and Natural History Museum Frankfurt, Clamecystrasse 12, 63571 Gelnhausen, Germany; 7 LOEWE Centre for Translational Biodiversity Genomics (LOEWE-TBG), Senckenberganlage 25, 60325 Frankfurt am Main, Germany; 8 CIBIO/InBio – Centro de Investigaҫao em Biodiversidade e Recursos Genéticos, Universidade do Porto, Campus Agrário de Vairão, 4485-661 Vairão, Portugal; 9 Departamento de Biologia, Faculdade de Ciências, Universidade do Porto, Rua do Campo Alegre s/n, 4169-007 Porto, Portugal; 10 Department of Zoology and Animal Cell Biology, University of the Basque Country (UPV/EHU), Paseo de la Universidad, 7, 01006 Vitoria-Gasteiz, Spain; 11 Department of Biology, Faculty of Veterinary Medicine, University of Zagreb, Heinzelova 55, 10000 Zagreb, Croatia; 12 Institute of Biodiversity and Ecosystem Research, Bulgarian Academy of Sciences, 2 Gagarin Street, 1113 Sofia, Bulgaria

**Keywords:** Genetics, Evolutionary biology, Genetic markers, Genomics

## Abstract

Intra- and inter-specific gene flow are natural evolutionary processes. However, human-induced hybridization is a global conservation concern across taxa, and the development of discriminant genetic markers to differentiate among gene flow processes is essential. Wolves (*Canis lupus*) are affected by hybridization, particularly in southern Europe, where ongoing recolonization of historic ranges is augmenting gene flow among divergent populations. Our aim was to provide diagnostic canid markers focused on the long-divergent Iberian, Italian and Dinaric wolf populations, based on existing genomic resources. We used 158 canid samples to select a panel of highly informative single nucleotide polymorphisms (SNPs) to (*i*) distinguish wolves in the three regions from domestic dogs (*C. l. familiaris*) and golden jackals (*C. aureus*), and (ii) identify their first two hybrid generations. The resulting 192 SNPs correctly identified the five canid groups, all simulated first-generation (F1) hybrids (0.482 ≤ *Q*_i_ ≤ 0.512 between their respective parental groups) and all first backcross (BC1) individuals (0.723 ≤ *Q*_i_ ≤ 0.827 to parental groups). An assay design and test with invasive and non-invasive canid samples performed successfully for 178 SNPs. By separating natural population admixture from inter-specific hybridization, our reduced panel can help advance evolutionary research, monitoring, and timely conservation management.

## Introduction

During the past centuries, long-term isolation and demographic declines in southern European peninsular populations of wolves (*Canis lup*us) caused considerable genetic drift that is now apparent in their genome-wide profiles^1–5^. However, the ongoing spatial and demographic recovery of most European wolf populations, mainly due to legal protection and increased prey and habitat availability^6,7^, has prompted a gradual process of natural contact with dispersal and gene flow among discrete populations^8–10^. This tendency is represented, for instance, by the wolf pack in Lessinia Regional Park in northern Italy, which was established in 2012 by a female from Italy and an immigrant male from the Dinaric Mountains in southwestern Slovenia near the border with Croatia^11,12^, followed by back-migration of their descendants to Slovenia detected during the national genetic wolf monitoring program^13^. Further examples are provided by the detection of Italian or Dinaric individuals re-sampled in the Iberian Peninsula^14^ and in southern Germany^15^.

However, despite growing numbers, several European wolf populations are now threatened by anthropogenic hybridization with free ranging domestic dogs (henceforth dogs, *C. l. familiaris*)^14,16,17^ and possibly with golden jackals (henceforth jackals, *C. aureus*)^18,19^. Wolf and jackal ranges are showing an increasing degree of overlap in several countries^20–22^, and hybridization between jackals and dogs has been confirmed^23^. The ability to rapidly and accurately discriminate natural population admixture from inter-specific hybridization is therefore especially important in areas where active interventions may be needed to mitigate anthropogenic hybridization, while allowing or facilitating inter-population gene flow^17,24–26^.

Earlier studies, prior to the development of genomic tools, provided key insights toward understanding population processes and wolf-dog hybridization based on traditional microsatellite loci (e.g., Ref.^27,28^). However, although multi-allelic microsatellites have a higher per-locus information content than bi-allelic single nucleotide polymorphism (SNP) markers and may have benefits for applications involving short spatiotemporal scales^29^, their use requires the calibration of results among laboratories when different datasets need to be merged. As an alternative, small panels of SNP markers, suitable also for non-invasive samples, have been developed for various species to address evolutionary and conservation questions (e.g., Ref.^30–34^). Although a larger number of SNPs are needed for tasks such as individual identification, SNPs permit high-throughput analyses where results are directly comparable among laboratories without the need for calibration—a major advantage when timely results are needed for cross-boundary conservation management decisions^32,33^.

von Thaden et al*.*^32^ performed comparative analyses for microsatellite and SNP data in wolves and European wildcats (*Felis silvestris*) where they noted that overall amplification rates were higher for SNPs than for microsatellites. However, new high-throughput sequencing approaches are providing important improvements also for microsatellite markers, as demonstrated for brown bears (*Ursus arctos*)^35^. These recent developments can permit the integration of different marker types and maximize the information obtained from non-invasive monitoring efforts. Because SNPs also permit insights into selective processes^36,37^, such markers can offer additional information for evolutionary research and may serve as indicators of responses to environmental change^38^.

Genomic methods are thus essential tools in wildlife conservation and management, and provide opportunities to track allele frequency changes in adaptive and deleterious loci for improved population monitoring (reviewed in Ref.^39^). SNP genotyping has also offered major advances for non-model species in fields such as biogeography^40^ and conservation genomics^41^. Modern genomic platforms provide extensive and cost-effective screening of thousands of SNPs, from which small panels of highly ancestry-informative markers (AIMs) can be selected depending on the specific research or monitoring questions at hand. These panels can be applied in extensive monitoring of endangered taxa or priority management units through microfluidic or quantitative PCR techniques^42^, which allow cost-effective genotyping of dozens of samples and markers at a time, including low quality DNA from non-invasively collected materials^32–34,43^, where multiple replicates can be performed when needed (see details in Ref.^32,33^).

A first set of ancestry-informative markers (AIMs) to accurately detect wolf *x* dog hybrids from non-invasively collected samples was recently identified by Harmoinen et al*.*^15^ and performed well for wolf populations across Europe. Their results confirmed the high genetic divergence among some southern European wolf populations, caused by protracted geographical isolation, demonstrated in earlier studies based on various genetic markers (e.g., Ref.^4,5,14,16,44^). Strong wolf population substructure and the increasing gene flow among wolf populations could therefore make individual assignment more difficult^3,17,27,44^. The 93-SNP panel proposed by Harmoinen et al*.*^15^ performs well for the distinct populations of Italy, Iberia, and the Dinaric region in detecting wolf *x* dog hybrids. The identification of additional highly informative SNPs can nevertheless help to distinguish natural admixture between wolves from these genetically divergent populations (e.g., Ref.^12^) from anthropogenic hybridization with dogs, a serious and increasing concern in all three regions^25,45,46^, and possible inter-specific hybridization with jackals (e.g., Ref.^23^). Building on the work of Harmoinen et al*.*^15^, we therefore selected an additional panel of highly informative SNPs. Our objectives were to: (*i*) identify dogs, jackals, and wolves from the Italian, Dinaric and Iberian populations in southern Europe and (*ii*) distinguish inter- and intra-specific first-generation hybrids (F1) and the first generation of backcrosses (BC1) with parental groups, to help prioritize conservation actions, especially in areas with high levels of anthropogenic hybridization^17,24,47,48^. Two additional objectives were to (*iii*) assess the SNP panel for the presence of loci under possible selection, and (*iv*) evaluate whether the SNP panel exhibits any clear bias in reconstructing wolf population divergence patterns, compared to existing results based on genome-wide profiles. These additional two assessments were performed to obtain a better understanding of the identified SNPs and their representation of the investigated wolf populations.

## Materials and methods

### Dataset building and filtering procedures

We combined existing SNP genotypes from wolves, dogs, and jackals typed on the Illumina CanineHD BeadChip including over 170,000 SNPs^2,49^ with unpublished data from Europe. European wolves included individuals from population ranges broadly defined in previous SNP analyses^1–3^ as Dinaric-Balkan (n = 112), Iberia (n = 25), and Italy (n = 77). Dog profiles included village dogs from Italy (n = 30), Croatia (n = 11) and Bulgaria (n = 2), considered more likely to hybridize with wolves^17,27,49^ or jackals^23^, and nine common dog breeds whose size is compatible with potential crossing with wolves (Bernese Mountain Dog—BMD, Border Collie—BoC, English Setter—ESt, Gordon Setter—GoS, Greyhound—GRe, German Shepherd—GSh, Labrador Retriever—LRe, Rottweiler—Rtw, and Weimaraner—Wei), genotyped by the LUPA project^50,51^. Jackal samples originated primarily from Bulgaria (n = 18), where possible hybridization between wolves and jackals have been reported^19^ and included samples from Croatia (n = 3) where hybrids with dogs have been documented^23^.

Canine genotypes mapped to the CanFam2 version of the dog genome were converted to the CanFam3 version with the UCSC liftOver tool (http://genome.ucsc.edu/cgi-bin/hgLiftOver) and imported into the SNP&Variant Suite 8.0.1 (hereafter SVS, Golden Helix Inc., Bozeman, MT). Nine of the SNPs included had previously been associated with three phenotypic traits (Table S1): black-coloured coat, white-coloured nails, and the presence of dewclaws, vestigial first toes on the hind legs^49,52^. In wolves, these traits have been linked to wolf *x* dog hybridization^49,53,54^, although it is important to note that they can also be found in individuals that do not exhibit any other sign of dog introgression^49^. These SNPs are henceforth described as phenotypic loci.

We performed an initial quality control of the dataset, by first applying a filter based on individual genotyping success and retaining profiles with ≥ 90% success rate. Next, we screened the data with a per-SNP genotyping threshold of > 90%. We then pruned the dataset for loci in strong linkage disequilibrium (LD) in SVS using the default settings: a LD-threshold of 0.5 and sliding windows of 50 SNPs. None of the nine phenotypic loci passed the initial filtering process, owing to missing values in the SNP data set. However, given their potential information value, we nevertheless selected three SNPs, one for each trait, for inclusion in the candidate panel (Table S1; selected loci in bold font). We next performed a preliminary assessment of all the canine profiles in Admixture 1.23^55^ with *K* = 3 population clusters (roughly corresponding to jackals, dogs and wolves) to determine overall population structure. To exclude possible hybrids, we ran pairwise comparisons, each time with *K* = 2, for each of the three wolf populations and dogs, then wolves and jackals, and finally dogs and jackals. We retained only wolves and jackals assigned with *q*_i_ ≥ 0.95 to their respective population clusters, and dogs assigned with *q*_i_ ≥ 0.90, considering their higher within-group variability.

### Marker selection

AIMs to identify admixture between dogs, jackals and the three regionally distinct wolf populations were selected by performing pairwise *F*_ST_ comparisons among the five canid groups and choosing loci with *F*_ST_ ≥ 0.90, evenly distributed across the 38 autosomal chromosomes. We then selected the 45 best-performing loci from each pairwise comparison for an initial panel. From this list, we selected a reduced panel by prioritizing loci that contributed to differentiation for (a) wolves *vs.* jackals, (b) *F*_ST_ values across pairwise comparisons for the three regional wolf populations, (c) Iberian *vs.* Italian wolves, (d) Italian *vs.* Dinaric wolves, (e) dogs *vs.* Italian wolves, (f) dogs *vs.* Iberian wolves, (g) dogs *vs.* Dinaric wolves, and (h) wolves *vs.* dogs across all three regions.

### Population structure analyses

We used the resulting SNPs in explorative multivariate analyses by performing a Discriminant Analysis of Principal Components (DAPC) in R 3.6.6^56^ with the Adegenet package 2.1.3^57^ to detect patterns of genetic differentiation among groups and individuals^58^. The “*find.clusters*” function was initially used to determine the best-supported number of genetic clusters using the Bayesian Information Criterion (BIC) running successive numbers (1–10) of *K*-means clusters of the individuals and the “*table.value*” function was used to graphically visualize the corresponding best clustering of the individuals. The optimal number of principal components (PCs) was identified using the “*optim.a.score*” function and the Discriminant Analysis (DA) was then run on the retained principal components using the “*dapc*” function. Finally, after selecting the optimal number of eigenvalues for the DA analysis, the DAPC results were graphically visualized with the “*scatter.dapc*” function and the assignment probability of individuals to each cluster were calculated and plotted using the “*assignplot*” function^59^.

We used the same dataset to perform Bayesian clustering analyses in Structure 2.3.4^60^. Five independent runs were performed for increasing values of *K* (the number of genetic clusters) from 1 to 10 using 500,000 Markov chain Monte Carlo (MCMC) iterations, after a burnin of 50,000 iterations, assuming no prior information (option *Usepopinfo* not activated), and choosing the “*Admixture*” and “*Independent Allele Frequency*” models^59^. We used the highest rate of increase in the posterior probability LnP(*K*) between consecutive values of *K* to estimate the most likely *K*-value, and then assessed the average (*Q*_i_) and individual (*q*_i_) proportions of membership in each cluster^61^. The software Clumpp 1.1.1^62^ was used to concatenate the data from the five independent runs for each *K* value, and Distruct 1.1^63^ to graphically display the results.

Subsequently, we used HybridLab 1.0^64^ to simulate jackal *x* wolf, wolf *x* dog, and wolf *x* wolf hybrids. The five canid groups were used as reference populations, and we simulated pairwise crosses to generate 10 F1-hybrids and 10 BC1-individuals with the wild parental populations for each comparison (Table S2). The resulting profiles were analyzed in Structure as described above to evaluate the efficiency of the selected SNP panel in detecting hybrids. Finally, we ran Adegenet (PC Analysis performed with the “*dudi.pca*” function) and Structure as described above with the 192 SNPs and a larger dataset of 191 individuals. This dataset included the five reference groups plus additional canids categorized as admixed or not admixed in earlier studies^23,26,28,46,49,65^ to test the discriminating power of the selected loci when applied to empirical data (comprising n = 10 Italian and n = 9 Dinaric wolves, n = 4 Italian wolf *x* dog, n = 4 Dinaric wolf *x* dog, n = 4 Iberian wolf *x* dog, and n = 2 jackal *x* dog hybrid individuals; admixed canids had been excluded from the marker selection step). To check the consistency of the inferred genetic structure, all clustering analyses were run again in Admixture to reassign each sample to its group of origin, assuming *K*-values from 1 to 10. The most likely number of clusters was identified based on the lowest cross-validation error^55^.

### Genetic variability analyses

The proportions of polymorphic (PL) and monomorphic (ML) loci observed and effective allele numbers (A_O_ and A_E_), observed and unbiased expected heterozygosity (H_O_ and uH_E_), numbers of private alleles (N_P_), Probabilities of Identity among unrelated (PID) and among full sib (PID_sibs_) individuals^66^, *F*_ST_ values, number of migrants (N_M_), and analysis of molecular variance (AMOVA) were computed using GenAlex 6.502^67^. The polymorphic information content (PIC) and the mean proportion of successfully genotyped loci (GL) were computed using Cervus 3.0.3^68^. Allelic richness (A_R_), which corrects the observed number of alleles for differences in sample sizes, was computed with Fstat 2.9.3.2^69^, and values of the inbreeding coefficient Wright’s *F*_IS_ were calculated using Genetix 4.03^70^.

### Candidate loci under selection and population divergence times

We evaluated the candidate SNPs for the possible presence of loci under selection (see full details in Supplemental Note S1). Finally, we used the candidate SNP loci to model estimated divergence times (in generations) among populations using various demographic scenarios (Supplemental Note S2).

### Fluidigm assay design, development and application

To verify the applicability of the resulting SNPs (see “Results”) in future genetic monitoring and conservation management, including their performance for non-invasively collected samples, we tested the SNPs on microfluidic 96 (samples) × 96 (loci) Dynamic Arrays™ (Fluidigm Corp., South San Francisco, USA), following Harmoinen et al.^15^. The SNP panel consisted of a) 84 SNPtype™ genotyping assays previously developed by Harmoinen et al.^15^ (Panel 1 in Table S5) and b) 108 newly designed SNPtype™ assays of the selected SNPs from this study (Panel 2 and Panel 3 in Table S5). We applied all 192 SNPtype™ genotyping assays to reference individuals previously genotyped on the Illumina CanineHD BeadChip, encompassing A) 33 DNA samples from muscular tissues, including 4 dogs, 7 jackals, 7 Dinaric, 7 Iberian, and 7 Italian wolves, plus one red fox (*Vulpes vulpes*) to check for other possible cross-species amplifications. Additionally, we included B) 60 non-invasive DNA samples obtained from faecal swabs^71^ including 11 Italian wolves, 4 dogs, and 45 hybrids previously identified using traditional microsatellites^26^. The samples in B) were collected during ongoing non-invasive genetic monitoring of the Italian wolf population. Finally, three blank controls were included. Invasive and non-invasive samples were genotyped 2–3 times and replicates were used to reconstruct consensus genotypes and calculate amplification success (henceforth AS) and error (Allelic Dropout, ADO and False Alleles, FA) rates in Gimlet 1.3.3^72^. Reliable Fluidigm consensus genotypes were then used to perform an additional Principal Coordinates Analysis (PCoA) using GenAlex 6.502^67^.

## Results

### Marker selection

Our initial filtering of the data retained 98,004 unlinked SNPs (including 3 phenotypic SNPs) available for a dataset of 158 canids, namely 52 dogs, 21 jackals (18 from Bulgaria and 3 from Croatia), and 30 Italian, 30 Dinaric, and 25 Iberian wolves. Following the selection process for highly discriminant loci, we retained an initial panel of 198 candidate SNPs (Table S3). These included 24 loci fixed in jackals, 13 fixed between dogs and Italian wolves, 4 fixed between dogs and Iberian wolves, 34 fixed between Italian and Iberian wolves and 7 fixed between Italian and Dinaric wolf populations (Table S3). From the preliminary panel of 198 SNPs, we then selected a reduced panel of 105 candidate SNPs (Table S4), comprising: (a) 15 loci discriminant for wolves and jackals (14 of which were fixed in jackals), (b) 37 SNPs common across *F*_ST_ tests for regional wolf populations, (c) 23 SNPs fixed between Italian and Iberian wolves, (d) 2 SNPs fixed between Italian and Dinaric wolves, (e) 13 SNPs fixed between dogs and Italian wolves, (f) 2 SNPs fixed between dogs and Iberian wolves, (g) 3 highly discriminant SNPs between dogs and Dinaric wolves and (h) 10 SNPs highly discriminant for wolf-dog tests in all the three geographic regions. These 105 SNPs were added to the three phenotypic loci (black-coloured coat, white-coloured nails, and the presence of dewclaws; Table S1) and to 84 of the 93 unlinked SNPs selected by Harmoinen et al*.*^15^ based on *F*_ST_ values between genome-wide profiles of European wolves and 58 dog breeds. Nine of the 93 SNPs from Harmoinen et al*.*^15^ did not pass the filtering process in our study and were removed. The total dataset for further analyses therefore comprised 158 canids and 192 SNPs highly informative across groups (Table S5).

### Population structure

**Figure 1:**
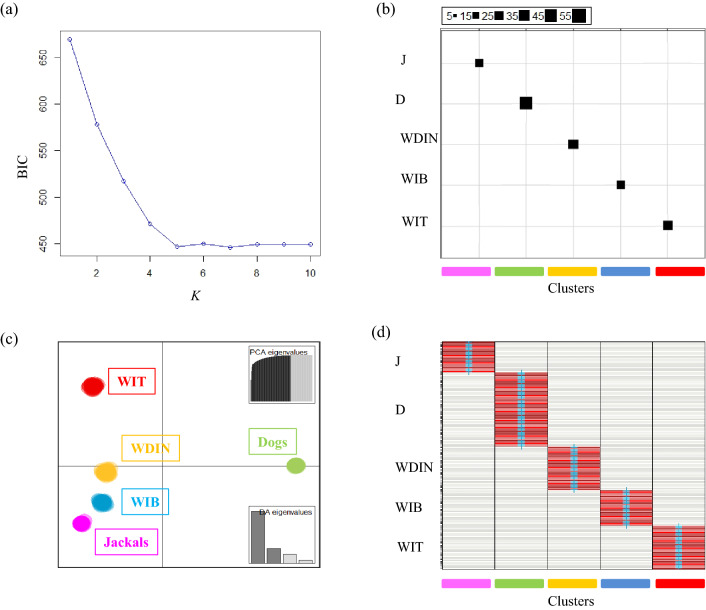
Explorative multivariate analyses performed with Adegenet 2.1.3^57^ on the 158 canid individuals typed at 192 SNPs. **(a)** Bayesian Information Criterion (BIC) run with successive numbers (from 1 to 10) of *K*-means clusters. The function “*table.value*” was used to graphically visualize the corresponding best clustering of the individuals. **(b)** Plot showing that the analysed individuals clustered in their five original sample groups which corresponded to the five inferred genetic clusters (J: Jackals, purple bar; D: domestic dogs, green bar; WDIN: Dinaric wolves, yellow bar, WIB: Iberian wolves, blue bar; WIT: Italian wolves, red bar). **(c)** Discriminant analysis of principal component (DAPC) scatterplot showing the genetic distribution among dogs (left side of PC-I) and wild canids (right side of PC-I, which explained 66% of the total genetic variability. PC-II (explaining 19% of the total genetic variability) shows the distinctions among the Italian wolves WIT (top of the graphic) and the other wolf (WIB: Iberian wolves, WDIN: Dinaric wolves) and jackal populations. See also the DA and PCA eigenvalue histogram inserted on the lower and upper right side, respectively. **(d)** Assignment plot based on DAPC: all individuals were assigned to their *a-priori* genetic clusters defined by the *K*-means analyses with individual membership probabilities > 0.999.

The *K*-means clustering analysis identified the best-supported number of genetic clusters at *K* = 5, which showed the lowest BIC value (Fig. 1a). At *K* = 5 all individuals clustered in their five original sample groups, which clearly corresponded to the five inferred genetic clusters (Fig. 1b). Discriminant analysis was performed using the five clusters defined by the *K*-means procedure, retaining the first 100 PCs during the data transformation step, which explained more than 99.5% of the total variance in the data, and four principal eigenvalues. The DAPC clearly identified the five original canid groups with the first component, explaining most of the genetic variability (*c.* 66%) and distinguished dogs from all of the wild canids, which were visibly separated along the second axis, describing *c.* 19% of the genetic diversity (Fig. 1c). Furthermore, the discriminant functions based on DAPC correctly assigned all individuals to their *a-priori* genetic clusters defined by the *K*-means analyses with individual membership probabilities always > 0.999 (Fig. 1d).

**Figure 2:**
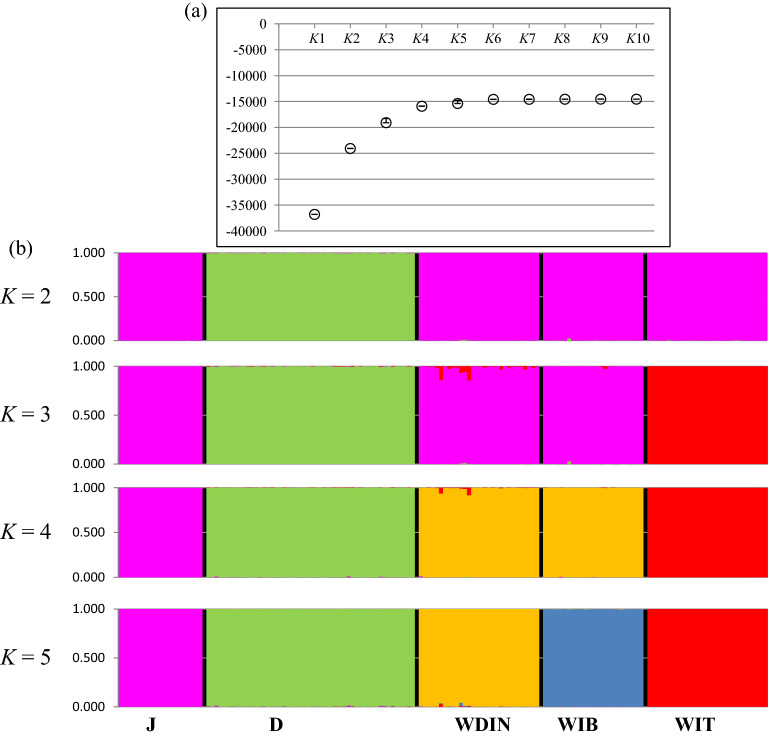
Results from the Bayesian assignment procedures. **(a)** Estimated posterior probability LnP(*K*) and corresponding standard deviations of the *K* clusters from 1 to 10. **(b)** Bar plots for the 158 individual *q*_i_-values obtained through assignment with the 192-SNP genotypes. Each individual is represented by a vertical line partitioned into coloured segments, whose length is proportional to the individual coefficients of membership (*q*_i_) in the jackal, dog and wolf clusters inferred by Bayesian assignment analyses performed in Structure2.3.4^60^, assuming *K* from 2 to 5, no prior information (option *Usepopinfo* not activated) and choosing the “*Admixture*” and “*Independent Allele Frequency*” models. J: Jackals; D: dogs; WDIN: Dinaric wolves, WIB: Iberian wolves; WIT: Italian wolves. Bar plots were obtained concatenating data from the five independent runs using Clumpp 1.1.1^62^ and graphically displayed using Distruct 1.1^63^.

Multivariate analyses were strongly supported by the Bayesian clustering procedures implemented in Structure that showed increasing rates in the estimated posterior probability LnP(*K*) of the clusters until *K* = 5 (Fig. 2a). For all *K*-values we observed very low standard deviations among different runs of the same *K*, with an average variation of only 0.00017 (± 0.00014 SD) in individual coefficient values (*q*_i_) among runs (Fig. 2a). At *K* = 2 (Fig. 2b), corresponding to the first main increase in LnP(*K*), dogs (mean estimated membership of population to the assigned cluster *Q*_1_ = 0.999) were clearly separated from the other four taxa (mean *Q*_2_ = 0.999). At *K* = 3, dogs (*Q*_1_ = 0.998) clustered separately from Italian wolves (*Q*_2_ = 1.000) and from the other wild canids (*Q*_3_ = 0.990). At *K* = 4, dogs were included in cluster 1 (*Q*_1_ = 0.997), Italian wolves in cluster 2 (*Q*_2_ = 0.999), jackals in cluster 3 (*Q*_3_ = 0.999), and Dinaric wolves together with Iberian wolves in cluster 4 (*Q*_4_ = 0.994). At *K* = 5 (Fig. 2b and Table 1), corresponding to the optimal number of genetic clusters and consistent with the phylogenetic and geographic subdivision of the samples, all five canid groups were correctly allocated in their own categories with dogs assigned to cluster 1 (*Q*_1_ = 0.996), Italian wolves to cluster 2 (*Q*_2_ = 0.999), jackals to cluster 3 (*Q*_3_ = 0.999), Dinaric wolves to cluster 4 (*Q*_4_ = 0.995) and Iberian wolves to cluster 5 (*Q*_5_ = 0.998). For *K* > 5, the LnP(*K*) reached a plateau and no further interpretable substructures were observed in the data (Fig. 2a). Notably, the substructure identified at the optimal genetic subdivision (*K* = 5) with the 192 SNPs was highly coherent with results obtained through an additional Structure analysis performed with the entire panel of 98,004 unlinked SNPs (Fig. S1a).

**Table 1:** Average membership proportions *Q*_i_ of the five canid groups in their original sampling group (in bold) with 90% confidence intervals (CI), estimated from the Bayesian assignment analyses performed in Structure 2.3.4^60^, assuming *K* = 5 clusters and using the “*Admixture*” and “*Independent allele frequencies*” models as parameter settings.

Populations	N	*Q*_J_ (90% CI)	*Q*_D_ (90% CI)	*Q*_WDIN_ (90% CI)	*Q*_WIB_ (90% CI)	*Q*_WIT_ (90% CI)
Jackals	21	**0.999 (0.995–1.000)**	0.000 (0.000–0.001)	0.000 (0.000–0.001)	0.000 (0.000–0.001)	0.000 (0.000–0.001)
Domestic dogs	52	0.001 (0.000–0.009)	**0.996 (0.977–1.000)**	0.001 (0.000–0.004)	0.001 (0.000–0.005)	0.001 (0.000–0.004)
Dinaric wolves	30	0.001 (0.000–0.004)	0.000 (0.000–0.002)	**0.995 (0.975–1.000)**	0.003 (0.000–0.012)	0.002 (0.000–0.008)
Iberian wolves	25	0.001 (0.000–0.003)	0.000 (0.000–0.001)	0.001 (0.000–0.004)	**0.998 (0.988–1.000)**	0.000 (0.000–0.001)
Italian wolves	30	0.000 (0.000–0.002)	0.000 (0.000–0.001)	0.000 (0.000–0.001)	0.000 (0.000–0.001)	**0.999 (0.994–1.000)**

Average *Q*_i_ were obtained concatenating the data from the five independent runs using Clumpp 1.1.1^62^.

**Figure 3:**
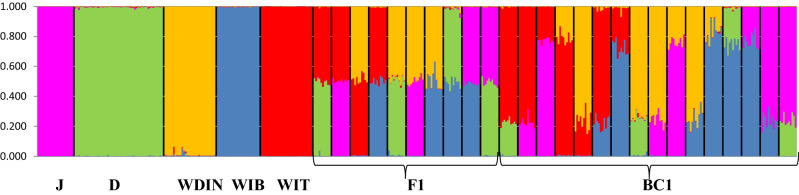
Bar plots for the 158 individual *q*_i_-values obtained through Structure 2.3.4^60^ assignment for the canid reference populations with the 192-SNP genotypes, assuming *K* = 5 clusters: jackals (J), dogs (D), Dinaric wolves (WDIN), Iberian wolves (WIB), Italian wolves (WIT), and 10 first-generation (F1) hybrid and first-generation backcross (BC1) genotypes simulated for each pairwise combination of canid groups. Bar plots were obtained concatenating data from the five independent runs using Clumpp 1.1.1^62^ and graphically displayed using Distruct 1.1^63^.

**Figure 4:**
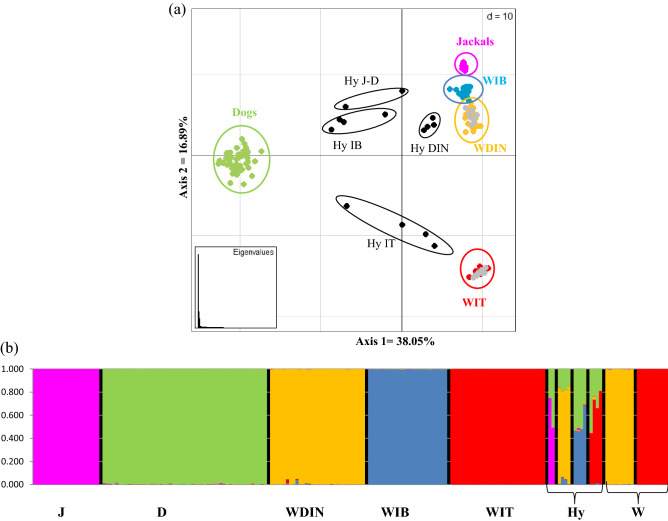
Multivariate discriminant analyses (PC performed with the “*dudi.pca*” function) **(a)** in the Adegenet 2.3.4^57^ package and **(b)** with a Bayesian assignment approach in Structure 2.3.4^60^ (assuming *K* = 5 and the “*Admixture*” and “*Independent allele frequencies*” models) obtained analysing the 192-SNP genotypes of the five reference populations (J: Jackals; D: Dogs; WDIN: Dinaric; WIB: Iberian; WIT: Italian wolves) together with 33 additional canid genotypes categorized as non-admixed (W, grey dots) or admixed (Hy, black dots) in earlier studies (n = 10 Italian and n = 9 Dinaric wolves, n = 4 Italian wolf *x* dog (HyIT), n = 4 Dinaric wolf *x* dog (HyDIN), n = 4 Iberian wolf *x* dog (HyIB), and n = 2 jackal *x* dog individuals (HyJ-D)). Bar plots were obtained concatenating data from the five independent runs using Clumpp 1.1.1^62^ and graphically displayed using Distruct 1.1^63^.

Bayesian clustering procedures run with the five canid groups plus their simulated first two generations of hybrids clearly confirmed that *K* = 5 corresponded to the optimal number of genetic clusters in the dataset with all five parental groups correctly assigned to their own cluster with 0.989 ≤ *Q*_i_ ≤ 0.998 (Fig. 3 and Table 2). As expected, all simulated F1 hybrids had intermediate *Q*_i_ assignment values (0.482 ≤ *Q*_i_ ≤ 0.512) between their respective parental groups (Fig. 3 and Table 2) and all BC1 showed *Q*_i_ assignment values to the parental wild groups they were backcrossed with ranging from 0.723 to 0.827 (Fig. 3 and Table 2). Finally, both multivariate discriminant analyses and Bayesian clustering procedures that were run with the additional 33 canid genotypes clearly demonstrated the high discriminating power of the identified loci when applied to empirical data. These correctly and unambiguously identified all wolf (WDIN *q*_i_ ≥ 0.992 and WIT *q*_i_ ≥ 0.999), all known wolf *x* dog (0.441 ≤ *q*_i_ ≤ 0.847) and jackal *x* dog (0.486 ≤ *q*_i_ ≤ 0.744) hybrid individuals  (Fig. 4a,b and Table S6). Bayesian clustering and assignment analyses run in Structure were highly concordant with the Maximum-likelihood clustering and assignment procedures performed in Admixture using both 192-SNP and 98,004-SNP parental population genotypes (Fig. S1b,c,d,e and Table S7, S8). Previous genome-wide analyses have indicated substructure between Dinaric and Balkan wolves^2^ and earlier studies from Bulgaria have reported wolf-dog and possibly also wolf-jackal hybridization^19^. We therefore performed additional tests with 10 wolf profiles from Bulgaria. These analyses indicated that F1-hybrids and BC1-individuals from the Balkan region can also be distinguished based on the identified loci (results not shown).

**Table 2:** Average membership proportions *Q*_i_ in their original sampling group (in bold) with 90% confidence intervals (CI) for the five canid groups and the first two generations of simulated hybrids, estimated from the Bayesian assignment analyses performed in Structure 2.3.4^60^, assuming *K* = 5 clusters and using the “*Admixture*” and “*Independent allele frequencies*” models as parameter settings.

	Populations	N	*Q*_J_ (90% CI)		*Q*_D_ (90% CI)		*Q*_WDIN_ (90% CI)		*Q*_WIB_ (90% CI)		*Q*_WIT_ (90% CI)	
Parentals	Jackals	21	**0.998**	**(0.990–1.000)**	0.000	(0.000–0.002)	0.001	(0.000–0.003)	0.001	(0.000–0.005)	0.000	(0.000–0.002)
	Domestic dogs	52	0.002	(0.000–0.014)	**0.992**	**(0.965–1.000)**	0.002	(0.000–0.013)	0.002	(0.000–0.015)	0.002	(0.000–0.011)
	Dinaric wolves	30	0.001	(0.000–0.008)	0.001	(0.000–0.005)	**0.989**	**(0.957–1.000)**	0.006	(0.000–0.030)	0.003	(0.000–0.014)
	Iberian wolves	25	0.001	(0.000–0.006)	0.000	(0.000–0.003)	0.002	(0.000–0.013)	**0.996**	**(0.978–1.000)**	0.001	(0.000–0.005)
	Italian wolves	30	0.000	(0.000–0.003)	0.000	(0.000–0.003)	0.001	(0.000–0.004)	0.001	(0.000–0.004)	**0.998**	**(0.989–1.000)**
Simulated F1	D-WIT	10	0.003	(0.000–0.017)	**0.492**	**(0.431–0.553)**	0.004	(0.000–0.025)	0.006	(0.000–0.028)	**0.496**	**(0.431–0.559)**
	J-WIT	10	**0.503**	**(0.431–0.574)**	0.000	(0.000–0.003)	0.002	(0.000–0.011)	0.002	(0.000–0.012)	**0.493**	**(0.422–0.565)**
	WIT-WDIN	10	0.002	(0.000–0.009)	0.001	(0.000–0.004)	**0.485**	**(0.395–0.574)**	0.005	(0.000–0.031)	**0.508**	**(0.422–0.594)**
	WIT-WIB	10	0.001	(0.000–0.006)	0.000	(0.000–0.003)	0.006	(0.000–0.039)	**0.501**	**(0.419–0.580)**	**0.492**	**(0.414–0.569)**
	D-WDIN	10	0.003	(0.000–0.018)	**0.502**	**(0.437–0.567)**	**0.482**	**(0.398–0.555)**	0.009	(0.000–0.054)	0.004	(0.000–0.027)
	J-WDIN	10	**0.500**	**(0.407–0.593)**	0.000	(0.000–0.002)	**0.496**	**(0.401–0.589)**	0.003	(0.000–0.020)	0.001	(0.000–0.006)
	WDIN-WIB	10	0.001	(0.000–0.008)	0.000	(0.000–0.003)	**0.496**	**(0.367–0.627)**	**0.501**	**(0.370–0.630)**	0.001	(0.000–0.008)
	D-WIB	10	0.007	(0.000–0.037)	**0.488**	**(0.424–0.552)**	0.010	(0.000–0.058)	**0.492**	**(0.408–0.564)**	0.004	(0.000–0.023)
	J-WIB	10	**0.512**	**(0.419–0.604)**	0.000	(0.000–0.002)	0.003	(0.000–0.015)	**0.485**	**(0.391–0.579)**	0.001	(0.000–0.004)
	D-J	10	**0.501**	**(0.436–0.564)**	**0.490**	**(0.430–0.552)**	0.003	(0.000–0.021)	0.004	(0.000–0.025)	0.002	(0.000–0.010)
Simulated BC1	D-WIT-WIT	10	0.002	(0.000–0.012)	**0.224**	**(0.175–0.275)**	0.004	(0.000–0.029)	0.004	(0.000–0.027)	**0.766**	**(0.710–0.816)**
	J-WIT-WIT	10	**0.226**	**(0.167–0.290)**	0.001	(0.000–0.003)	0.002	(0.000–0.010)	0.002	(0.000–0.010)	**0.770**	**(0.707–0.829)**
	J-WIT-J	10	**0.772**	**(0.709–0.831)**	0.000	(0.000–0.002)	0.002	(0.000–0.011)	0.002	(0.000–0.012)	**0.224**	**(0.166–0.286)**
	WIT-WDIN	10	0.002	(0.000–0.010)	0.001	(0.000–0.003)	**0.213**	**(0.137–0.291)**	0.005	(0.000–0.035)	**0.780**	**(0.705–0.849)**
	WIT-WDIN	10	0.001	(0.000–0.007)	0.000	(0.000–0.003)	**0.763**	**(0.680–0.838)**	0.004	(0.000–0.025)	**0.232**	**(0.158–0.311)**
	WIT-WIB-WIT	10	0.001	(0.000–0.008)	0.000	(0.000–0.003)	0.013	(0.000–0.081)	**0.226**	**(0.130–0.303)**	**0.760**	**(0.689–0.826)**
	WIT-WIB-WIB	10	0.001	(0.000–0.008)	0.000	(0.000–0.003)	0.014	(0.000–0.070)	**0.723**	**(0.638–0.798)**	**0.261**	**(0.192–0.334)**
	D-WDIN-WDIN	10	0.003	(0.000–0.017)	**0.247**	**(0.194–0.304)**	**0.737**	**(0.661–0.798)**	0.008	(0.000–0.051)	0.005	(0.000–0.027)
	J-WDIN-WDIN	10	**0.240**	**(0.161–0.325)**	0.000	(0.000–0.003)	**0.754**	**(0.666–0.835)**	0.004	(0.000–0.026)	0.001	(0.000–0.010)
	J-WDIN-J	10	**0.756**	**(0.674–0.833)**	0.000	(0.000–0.003)	**0.240**	**(0.162–0.323)**	0.003	(0.000–0.017)	0.001	(0.000–0.006)
	WDIN-WIB-WDIN	10	0.001	(0.000–0.009)	0.000	(0.000–0.004)	**0.746**	**(0.619–0.864)**	**0.250**	**(0.132–0.377)**	0.002	(0.000–0.013)
	WDIN-WIB-WIB	10	0.001	(0.000–0.008)	0.000	(0.000–0.003)	**0.167**	**(0.054–0.286)**	**0.827**	**(0.711–0.927)**	0.004	(0.000–0.027)
	D-WIB-WIB	10	0.002	(0.000–0.014)	**0.243**	**(0.191–0.298)**	0.005	(0.000–0.030)	**0.746**	**(0.682–0.803)**	0.004	(0.000–0.026)
	J-WIB-WIB	10	**0.226**	**(0.148–0.311)**	0.000	(0.000–0.002)	0.002	(0.000–0.012)	**0.771**	**(0.685–0.850)**	0.001	(0.000–0.004)
	J-WIB-J	10	**0.758**	**(0.675–0.834)**	0.000	(0.000–0.002)	0.002	(0.000–0.014)	**0.239**	**(0.162–0.322)**	0.001	(0.000–0.004)
	D-J-J	10	**0.758**	**(0.704–0.807)**	**0.237**	**(0.189–0.288)**	0.002	(0.000–0.013)	0.002	(0.000–0.012)	0.001	(0.000–0.008)

Data comprise the 192-SNP genotypes of the parental populations (jackals, dogs, Dinaric, Iberian and Italian wolves), their first-generation hybrids (F1) and the first backcross (BC1) of simulated admixed genotypes. J = jackal, D = dog, W = wolf, IT = Italy, DIN = Dinaric, IB = Iberia. Average *Q*_i_ were obtained concatenating the data from the five independent runs using Clumpp 1.1.1^62^.

### Genetic variability

The proportions of polymorphic loci ranged from about 8% in jackals to more than 80% in dogs, with intermediate values (30%-45%) in the three wolf populations (Table 3). The highest rates of A_O_, A_E_, A_R_, PIC, H_O_ and uH_E_ were observed in dogs and the lowest values were seen in jackals (Table 3). Among the wolf populations, Italian wolves showed the lowest values for the variability indexes, followed by Iberian and Dinaric wolves (Table 3). AMOVA showed that more than 81% of the total genetic diversity was significantly (*P* < 0.001) partitioned among the five groups. All pairwise *F*_ST_ values (Table 4) were highly statistically significant (*P* < 0.0001) and aligned with the very low corresponding effective *F*_ST_-based migration rates (Table 4). The PID and PID_SIBS_ values for the 192 loci, and the probability of finding multiple individuals with the same genotype, were low (Table 3).

**Table 3:** Genetic variability at 192 SNPs in jackals, dogs, and wolves from the Dinaric, Iberian and Italian populations.

Populations (N)	GL (%)	PL (%)	ML (%)	A_O_	A_E_	A_R_	N_P_	PIC	H_O_	uH_E_	*F*_IS_	*P*	PID	PID_sibs_
Jackals (21)	99.581	8.334	91.666	1.083 (0.020)	1.039 (0.012)	1.076	2	0.019	0.024 (0.007)	0.023 (0.007)	− 0.035	0.240	1.343 × 10^–04^	1.039 × 10^–02^
Domestic dogs (52)	99.712	88.545	11.455	1.885 (0.023)	1.481 (0.022)	1.859	31	0.239	0.264 (0.010)	0.296 (0.011)	0.110	< 0.001 ***	1.005 × 10^–50^	4.279 × 10^–26^
Dinaric wolves (30)	99.861	46.352	53.648	1.464 (0.036)	1.156 (0.020)	1.383	0	0.081	0.098 (0.012)	0.099 (0.011)	0.010	0.348	2.776 × 10^–18^	3.828 × 10^–09^
Iberian wolves (25)	99.793	35.946	64.054	1.359 (0.035)	1.114 (0.017)	1.322	0	0.063	0.065 (0.009)	0.076 (0.010)	0.143	< 0.001 ***	2.002 × 10^–13^	3.855 × 10^–07^
Italian wolves (30)	99.913	32.818	67.182	1.328 (0.034)	1.092 (0.017)	1.245	0	0.047	0.058 (0.010)	0.057 (0.009)	− 0.003	0.439	2.428 × 10^–10^	1.247 × 10^–05^

Values in parentheses denote standard errors. N = sample size; GL = percentage of mean proportion of successfully genotyped loci; PL = proportion of polymorphic loci; ML = proportion of monomorphic loci; A_O_ = number of observed alleles; A_E_ = number of expected alleles; A_R_ = allelic richness; N_P_ = number of private alleles; PIC = polymorphic information content; H_O_ = observed heterozygosity; uH_E_ = unbiased expected heterozygosity; *F*_IS_ = estimates of departure from Hardy–Weinberg equilibrium;* P* = probability of obtaining, by chance, *F*_IS_ values higher than those observed after 10,000 random permutations (*P < 0.05, **P < 0.01, ***P < 0.001); PID = probability of identity; PID_sibs_ = probability of identity among full sibs.

**Table 4:** Matrix of pairwise *F*_ST_ values (below the diagonal) and the corresponding estimates of historical gene flow N_M_ (above the diagonal) among the sampled populations computed using 192-SNP genotypes.

Populations	Jackals	Domestic dogs	Dinaric wolves	Iberian wolves	Italian wolves
Jackals	−	0.119	0.091	0.070	0.033
Domestic dogs	0.677	−	0.141	0.136	0.110
Dinaric wolves	0.734	0.640	−	0.257	0.087
Iberian wolves	0.781	0.648	0.493	−	0.058
Italian wolves	0.884	0.695	0.741	0.813	−

All *F*_ST_ values were highly statistically significant (P < 0.0001).

### Candidate loci under selection and population divergence times

With a false discovery rate threshold of 0.05, BayeScan^73^ identified n = 3 putative outlier SNPs, one for each pairwise comparison between wolf populations, mapped on chromosomes 1, 8, and 25, respectively (See Supplemental Note S1, Fig. S2 and Fig. S3 for details). For wolf population divergence times, ABC simulations showed the highest support for scenario number 7 (sequential population splitting with subsequent bottlenecks), which performed better than the other models (Supplemental Note S2).

### Fluidigm assay design, development and application

**Figure 5:**
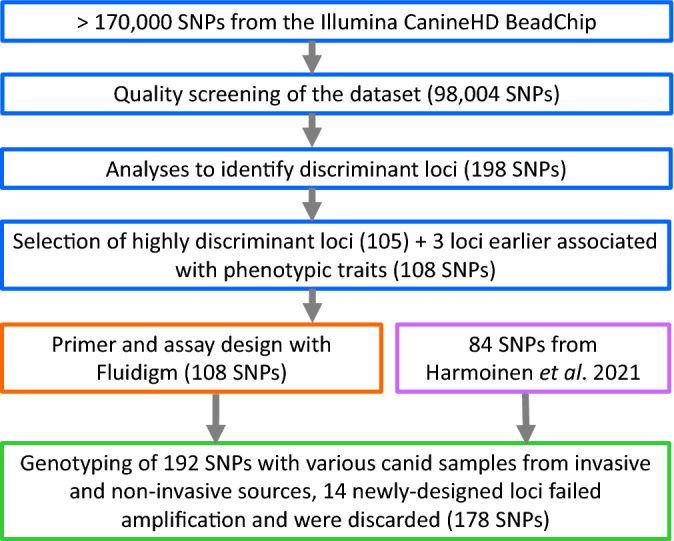
Flowchart describing the SNP selection process for design of the reduced marker panel.

We successfully designed primer pairs and assays for all the new 108 SNPs that satisfied the recommended parameters (minimum separation distance of 100 base pairs), avoiding regions with large repeats or otherwise difficult for the interpretation of results. After amplifying the 93 DNA samples 2–3 times at the 192 SNPs, we discarded 14 loci among the newly-designed SNPs (corresponding to 13% of panel 2–3, and 7% of the 192 SNPs) that failed amplifications in all reactions (Tables S3, S4, and S5), resulting in 178 SNPs (Fig. 5). Genotyping success for the remaining 178 SNPs was high across samples and markers, and only one non-invasively sampled dog with 96% missing data was removed from the analyses. All of the remaining wolf, hybrid, and dog samples were successfully genotyped at ≥ 95% of the tested loci, showing an average amplification success rate of 98% and an average allelic dropout rate of 4.4% across loci (Table S5). As expected, invasive samples showed the highest AS (99%) and the lowest error (0.7%) rates (Table S5), and their genotypes fully matched the original profiles from the Illumina CanineHD BeadChip. Interestingly, non-invasive samples showed only slightly lower AS (97%) and only slightly higher error (3.7%) rates across loci (Table S5). Cross-species amplification tests also produced valid genotypes for the other canid species with jackals showing AS = 97%, ADO = 1% and FA = 0, and red foxes showing AS = 90%, ADO = 0.5% and FA = 0 (Table S5). Notably, Panel 1 confirmed its high performance (AS = 99%, ADO = 0.6% and FA = 0 for tissue samples and AS = 98%, ADO = 6.1% and FA = 0 for faecal samples) for genotyping of various DNA sources. The newly designed and tested Panels 2 and 3 also performed well for genotyping both invasive (AS = 98%, ADO = 0.8% and FA = 0) and non-invasive samples (AS = 96%, ADO = 1.6% and FA = 0) on the Fluidigm platform (Table S5). A PCoA on invasive and non-invasive samples genotyped with the 178 SNPs confirmed the previous assignments. The different canid groups were clearly separated, with non-invasive genotypes clustering with profiles from their respective reference groups (Fig. S4). Admixed individuals were placed between parental groups, with recent admixture resulting in intermediate positions and older backcrosses positioned closer to wild parental groups (Fig. S4). Importantly, re-analyses with 178 of 192 SNPs did not affect the overall results. Outcomes from multivariate, Bayesian and Maximum-likelihood assignment and clustering analyses in Adegenet, Structure and Admixture with the five reference populations and 178-SNP genotypes (Fig S5a; Table S9 and S12), simulated (Fig S5b; Tables S10 and S13) and other empirical data (Fig S5c,d; Table S11), were always significantly correlated (*R* ≥ 0.997; *P* < 0.0001 for all pairwise combinations) and not significantly different (*t* ≥ 0.005, *P* ≥ 0.977; *t*-tests for all pairwise combinations) from the results obtained with 192 SNPs.

## Discussion

We employed a reduced panel of 192 SNPs to reliably discriminate between anthropogenic hybridization and natural gene flow among populations in five canid groups, centered on wolves in southern Europe. Of these SNPs, 178 were successfully amplified on the Fluidigm platform, and provided the same results as the 192 SNPs for analysis of population structure. Results from empirical data and simulated profiles suggest that the selected marker set is well-suited for this purpose, and our analyses unambiguously identified all F1-hybrids and all BC1-individuals. Our results have shown that the panel performs well, also for non-invasively collected samples, and the reduced set of SNPs presented by Harmoinen et al.^15^ and in this study can therefore provide informative results for monitoring, research, and conservation management.

### Marker selection and the power of discriminant loci

Our results based on empirical data and simulated profiles indicated that the identified SNPs clearly differentiate F1-hybrids and accurately detect BC1-individuals within and across canid species, showing no type I (non-admixed individuals erroneously identified as admixed animals) nor type II (admixed individuals falsely identified as non-admixed animals) errors^26^. The identified loci incorporate the panel of 93 SNPs developed for microfluidic genotyping by Harmoinen et al*.*^15^, who found almost identical genotypes in a test of 30 invasive and non-invasive samples from the same wolves. As a complement to the European wolf-dog hybrid panel presented by Harmoinen et al*.*^15^, we therefore propose the selected SNPs for a more detailed evaluation of population admixture *versus* hybridization in southern European populations, including possible events of jackal-hybridization^19,23^.

The presence of fixed alleles in jackals, dogs, and between wolf populations contributed to the discriminant power of certain loci, which can be particularly valuable as diagnostic markers for monitoring purposes. Where incomplete profiles are obtained from non-invasive samples, the results from a few such markers can be enough to allow species identification^74^, which is highly relevant for tracking the expanding distribution of jackals. Their rapid range expansion across Europe^22^ and within our study area, recently illustrated by the confirmation of jackal presence south of the Po River in northern Italy (http://gojage.blogspot.com/2021/01/all-roads-lead-to-rome-european-golden.html), highlights the urgent need to account for this species in future canid monitoring and management plans. Our results indicated that the selected SNPs perform well for identification of F1-hybrids and BC1-individuals for all canid group combinations. Detecting the presence and distribution of these early-generation hybrids is typically a higher priority than that of individuals with lower proportions of dog ancestry, to prioritize scarce conservation resources^26^. The findings from our empirical and simulated data therefore indicate that our panel can reliably identify the animals that are most essential for monitoring and conservation management (e.g., the LIFE MIRCO-Lupo project, http://www.lifemircolupo.it/).

Although the original panel of 93 SNPs from Harmoinen et al*.*^15^ was developed and successfully tested with non-invasive samples, and the additional loci presented in this study also performed well with non-invasive sources of DNA, we underline that the proposed panel may need future modifications given the highly dynamic situation for canids in our study area. In view of the recent gene flow between Italian Alpine and Dinaric wolves^12^, the detection of dog-jackal hybridization^23^ and the increasing overlap seen in wolf and jackal distributions throughout Europe^21^, it will also be necessary to monitor for possible changes in the spatial and temporal distribution of allele frequencies in the selected SNPs^26^. However, ongoing international collaborations in population monitoring, as illustrated by the LIFE WolfAlps EU project (http://www.lifewolfalps.eu/), will provide such information for wolves and other wide-ranging carnivore species. Accordingly, the opportunities for data sharing offered by new genomic approaches will contribute toward keeping these tools as useful and updated as possible.

### Population structure

All five *a priori* sampling groups emerged as well-separated genetic clusters based on multivariate and Bayesian clustering analyses of the identified SNPs. As expected, dogs represented the most divergent group, and Italian wolves were also clearly differentiated from the other wolves, as observed in earlier analyses of SNP profiles^1–3^ and supported by earlier findings from analyses of mitochondrial DNA and microsatellite markers^11,75,76^. Moreover, pairwise values of genetic distances between populations align with previous findings^1–3^ for southern European wolf populations. Notably, jackals emerged as a distinct group only at *K* = 4 population clusters, which is likely a reflection of the ascertainment bias in the SNP panel originally developed for dogs^1^.

### Genetic variability

The lower diversity values for Italian wolves, followed by Iberian wolves and Dinaric wolves, accord with earlier SNP analyses of European wolf populations^1–3^. It is nonetheless important to note that the reported levels of diversity differ among analyses, and that the *a priori* selection of loci for the purposes of our study likely influenced the results. The SNP array that formed the basis of our study is centered on genetic variation in dogs^1,77^, magnifying their genetic diversity while underestimating that of wolves and the more distantly related jackals^1^. Furthermore, our selection of markers focused on SNPs with high *F*_ST_-values. For these reasons, direct comparisons of genetic variability among taxa should be treated with caution. Future developments based on whole-genome data may provide additional new markers less affected by ascertainment bias. However, the selection of loci from the existing—and commonly used—SNP panel facilitates the use of already-published canid profiles from earlier projects as reference populations, which could increase the utility of the panel.

Notwithstanding, we report various diversity parameters for comparative purposes, which might also inform future efforts for different canid groups or other taxa. Relevant examples are the values for PID and PID_sibs_, where values calculated across 21 jackals (PID = 1.343 × 10^–04^, PID_sibs_ = 1.039 × 10^–02^) were substantially higher than those calculated for the 30 Italian wolves (PID = 2.428 × 10^–10^, PID_sibs_ = 1.247 × 10^–05^) that represented the 2nd-highest values. Nonetheless, our selected SNPs provide essential information, given that the initial 93-SNP panel by Harmoinen et al*.*^15^ did not distinguish among wild canids (i.e., among wolves, jackals, and red foxes). Although a higher number of SNPs are required to obtain PID values similar to standard microsatellite panels^32,43^, careful testing have shown that such panels can still work well across broad geographic regions and divergent populations^15^.

### Candidate loci under selection and population divergence times

We detected a limited number of loci under possible selection (Supplemental Note S1). Although earlier SNP analyses have suggested possible environmental selection in European wolves^3,78^, future research with more samples and genomic data are needed to evaluate these findings, including the potential presence of false positives among our results. Our population divergence modeling (Supplemental Note S2) indicated sequential population splits and bottlenecks broadly consistent with whole-genome results^5^, although our results showed very wide confidence intervals and could be partly affected by ascertainment bias, mutation rates, marker numbers, and secondary gene flow^3,5,79^.

### Fluidigm assay design, development and application

Our results indicate that the selected SNPs are well-suited for conservation management, including projects based on non-invasively collected samples. When tested on standard platforms such as the microfluidic arrays, most of the candidate markers performed efficiently and reliably, showing high amplification success and low error rates on various DNA sources from different canid groups. Well-differentiated allele frequencies allowed us to distinguish among the different canid groups and their first generation hybrids. Importantly, we were also able to distinguish the aforementioned groups from the red fox, whose DNA may be present in non-invasively collected samples such as livestock damage cases.

### Conclusion and recommendations

We found that the identified SNPs can reliably distinguish natural population admixture from inter-specific hybridization in five canid groups comprising dogs, jackals, and the long-divergent Iberian, Italian and Dinaric wolf populations, up to the first generation of backcrosses, using various sources of DNA, including non-invasively collected materials. New genomic tools provide opportunities to integrate data from different laboratories without the need for calibration, which is essential for wide-ranging species such as wolves. The sequencing of microsatellite markers^35^, which is now in progress for wolves (Skrbinšek et al., *unpublished data*), may also offer opportunities to combine hypervariable microsatellites with SNP loci possibly associated with adaptive genetic variation. The selected SNPs can provide timely identification of dispersers and ensure that such important individuals are not erroneously classified as hybrids, with the subsequent risk of management decisions that could be harmful for conservation. The proposed panel will also facilitate the monitoring of jackals, which are rapidly expanding their ranges across Europe, and contribute to research, monitoring, and conservation management of wild European canids.

## Supplementary Information

Supplementary Information 1.

Supplementary Information 2.

Supplementary Information 3.

Supplementary Information 4.

Supplementary Information 5.

Supplementary Information 6.

Supplementary Information 7.
