# Supplementary figures and images for: A reduced SNP panel to trace gene flow across southern European wolf populations and detect hybridization with other Canis taxa
Source: Sci Rep. 2022 Mar 9;12:4195. doi: 10.1038/s41598-022-08132-0 (PMC8907317; doi:10.1038/s41598-022-08132-0)

## STRUCTURE analyses with 98k SNPs

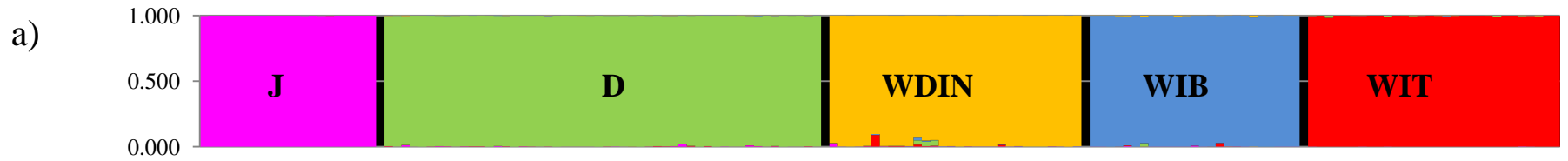

## ADMIXTURE analyses with 98k SNPs

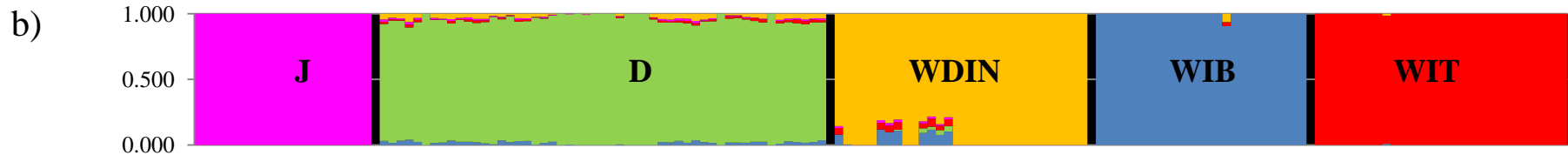

## ADMIXTURE analyses with 192 SNPs

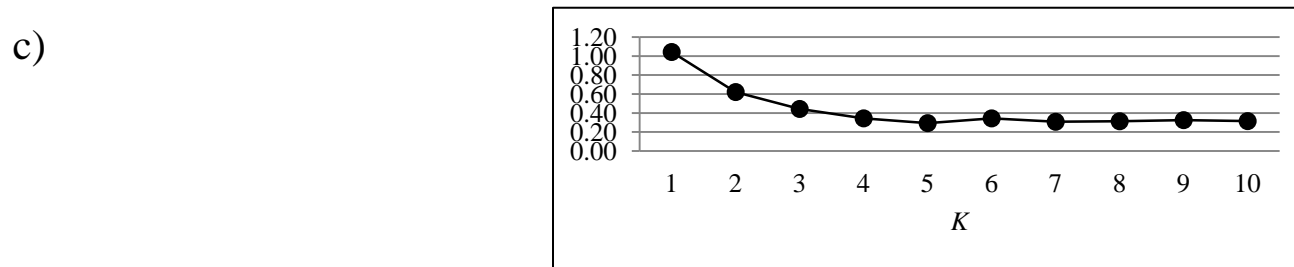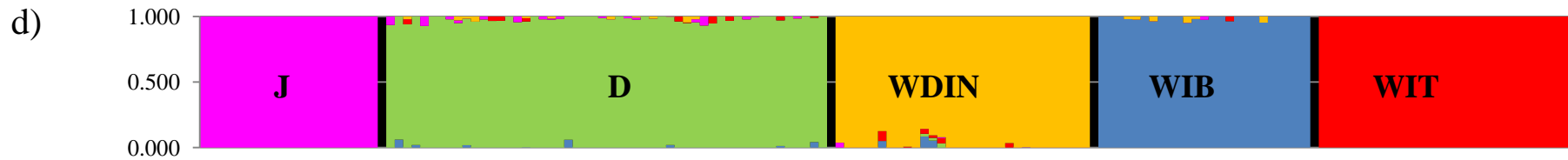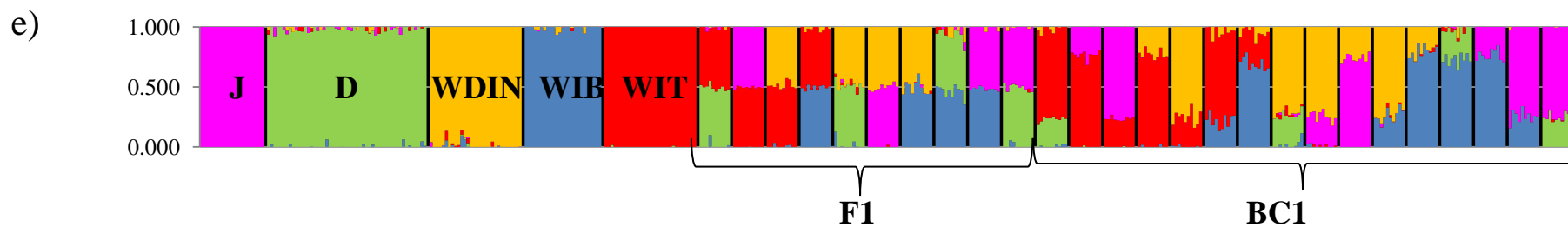

Supplement: Supplementary file 1 — Supplementary Information 1. [file 41598_2022_8132_MOESM1_ESM.pdf]

## Scenario 7

- N2b
- N3b
- N1b
- NA
- N1
- N2
- N3
- N4

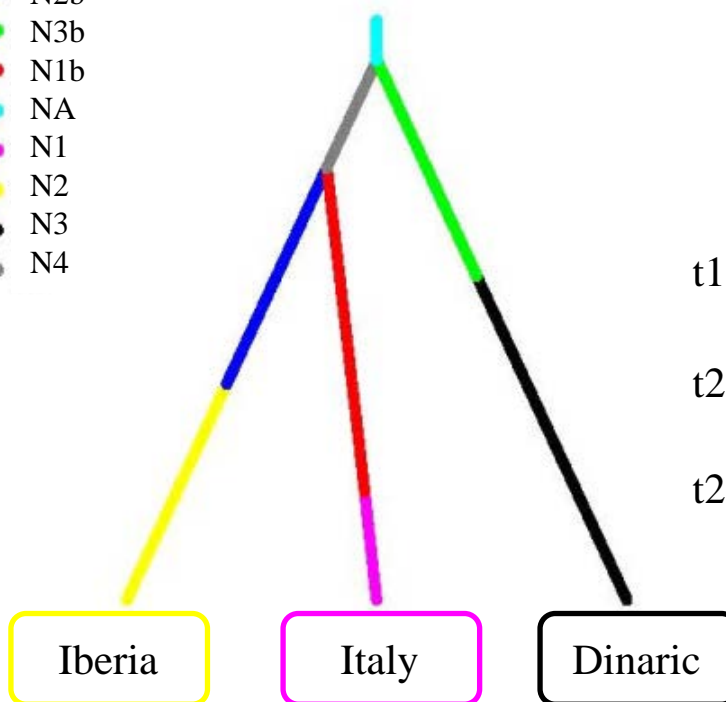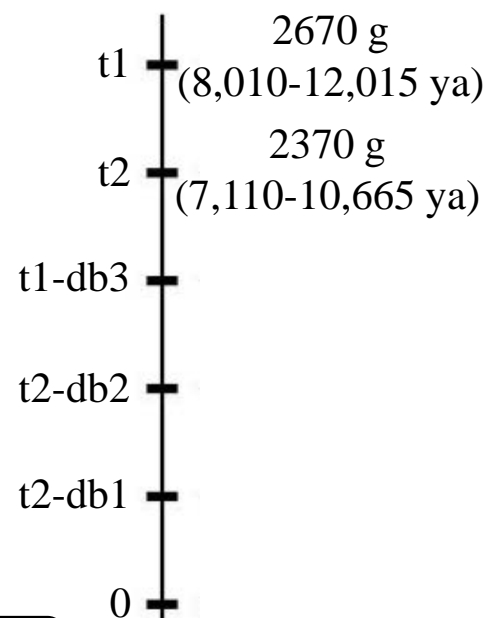

Supplement: Supplementary file 3 — Supplementary Information 3. [file 41598_2022_8132_MOESM3_ESM.pdf]

# Principal Coordinates Analysis (PCoA) with 178 SNPs

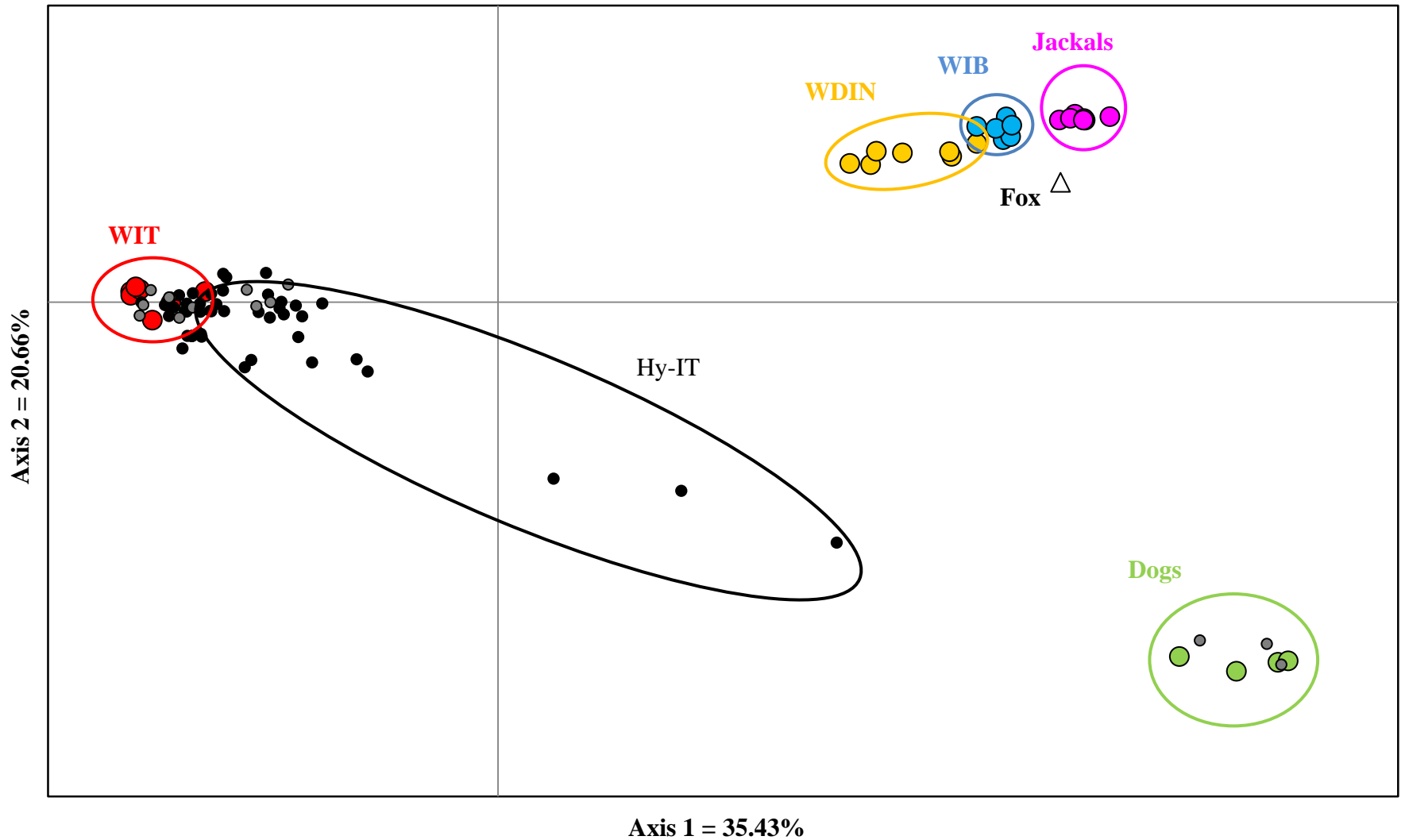

Supplement: Supplementary file 4 — Supplementary Information 4. [file 41598_2022_8132_MOESM4_ESM.pdf]

Bayesian and multivariate analyses with 178 SNPs

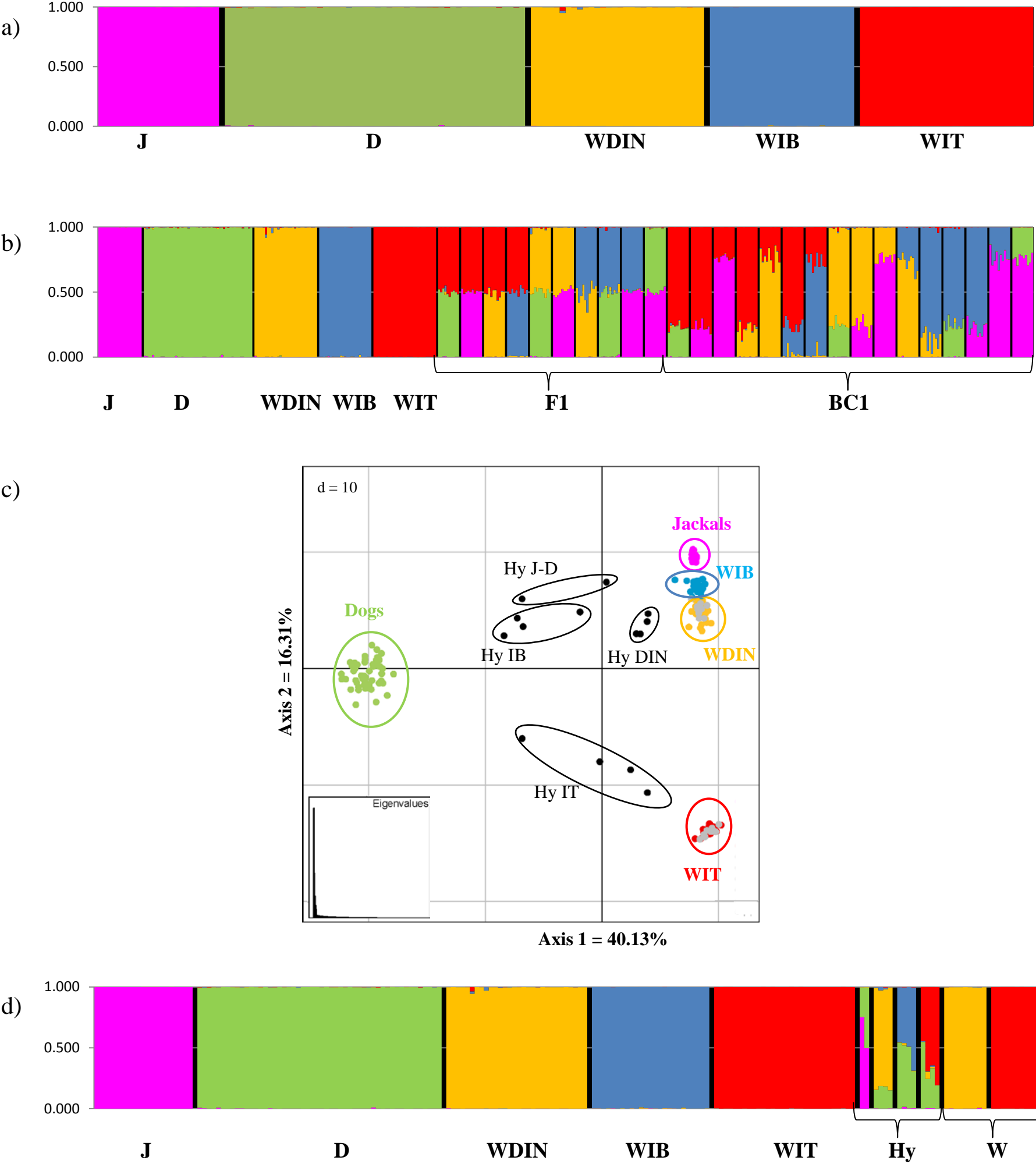

Supplement: Supplementary file 5 — Supplementary Information 5. [file 41598_2022_8132_MOESM5_ESM.pdf]
