# Supplementary material for: A reduced SNP panel to trace gene flow across southern European wolf populations and detect hybridization with other Canis taxa
Source: Sci Rep. 2022 Mar 9;12:4195. doi: 10.1038/s41598-022-08132-0 (PMC8907317; doi:10.1038/s41598-022-08132-0)
Supplement: Supplementary file 2 — Supplementary Information 2. [file 41598_2022_8132_MOESM2_ESM.pdf]

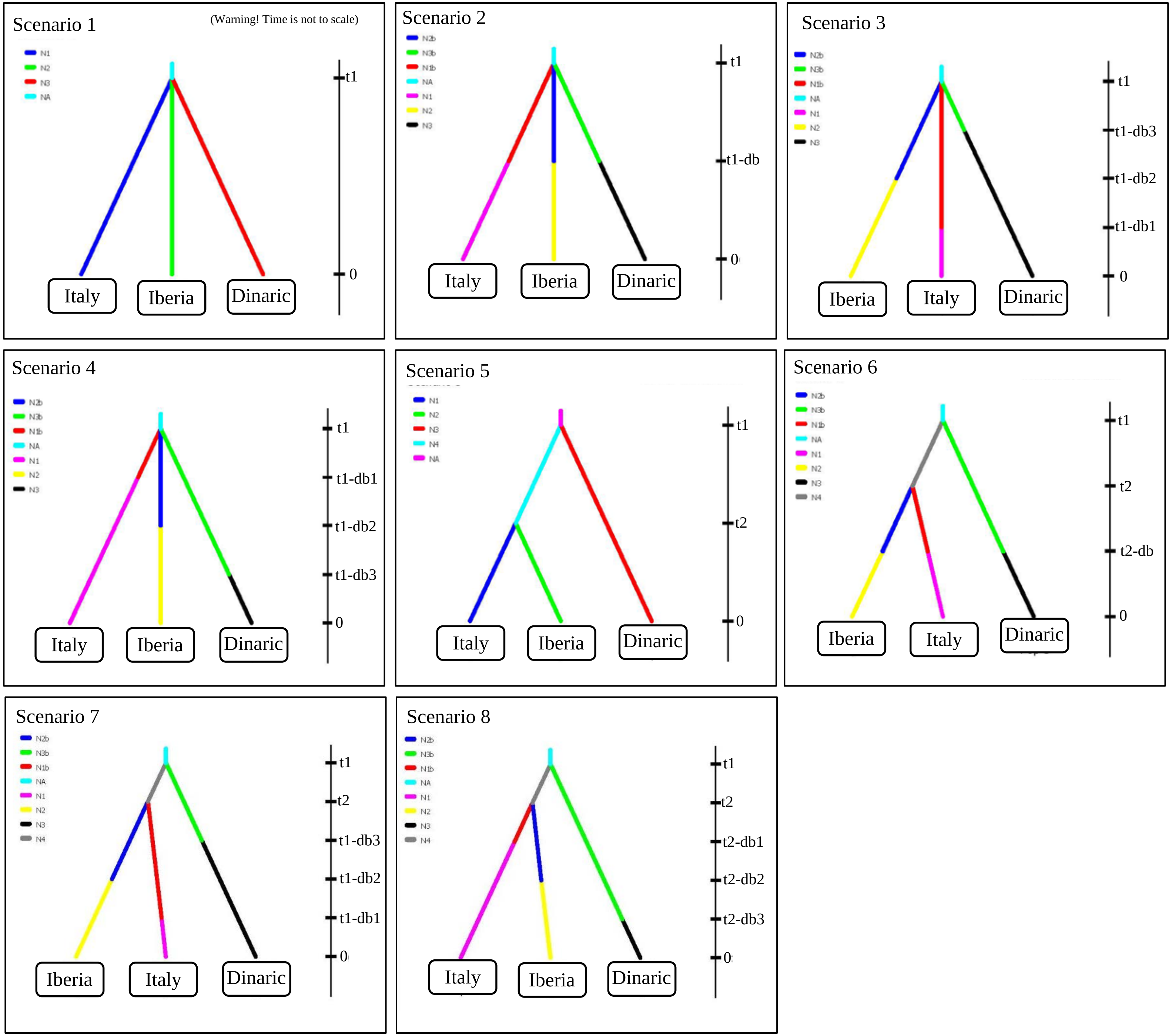
Scenario 1
(Warning! Time is not to scale)
N1
N2
N3
NA
t1
0
Italy
Iberia
Dinaric
Scenario 2
N2b
N3b
N1b
NA
N1
N2
N3
t1
t1-db
0
Italy
Iberia
Dinaric
Scenario 3
N2b
N3b
N1b
NA
N1
N2
N3
t1
t1-db3
t1-db2
t1-db1
0
Iberia
Italy
Dinaric
Scenario 4
N2b
N3b
N1b
NA
N1
N2
N3
t1
t1-db1
t1-db2
t1-db3
0
Italy
Iberia
Dinaric
Scenario 5
N1
N2
N3
N4
NA
t1
t2
0
Italy
Iberia
Dinaric
Scenario 6
N2b
N3b
N1b
NA
N1
N2
N3
N4
t1
t2
t2-db
0
Iberia
Italy
Dinaric
Scenario 7
N2b
N3b
N1b
NA
N1
N2
N3
N4
t1
t2
t1-db3
t1-db2
t1-db1
0
Iberia
Italy
Dinaric
Scenario 8
N2b
N3b
N1b
NA
N1
N2
N3
N4
t1
t2
t2-db1
t2-db2
t2-db3
0
Italy
Iberia
Dinaric
