# Supplementary material for: A reduced SNP panel to trace gene flow across southern European wolf populations and detect hybridization with other Canis taxa
Source: Sci Rep. 2022 Mar 9;12:4195. doi: 10.1038/s41598-022-08132-0 (PMC8907317; doi:10.1038/s41598-022-08132-0)
Supplement: Supplementary file 6 — Supplementary Information 6. [file 41598_2022_8132_MOESM6_ESM.docx]

Supplemental materials for “**A reduced SNP panel to trace gene flow across southern European wolf populations and detect hybridization with other *Canis* taxa**”

**Supplemental Note S1. Identification of candidate loci under selection**

*Methods.* To identify candidate SNPs under selection, the *F*_ST_-based approach implemented in the program BayeScan 2.1^1^, which detects loci where allele frequencies differ significantly from the average genome-wide distances between populations, was run using default parameters of 5,000 iterations with a burn-in length of 100,000, prior odds of 10 and a conservative false discovery rate (the expected proportion of false positives) of 0.05. Subsequently, we selected an interval of 100 Kb on each side of every outlier SNP^2-3^ and used the coordinates on the CanFam3.1 reference assembly to i) retrieve the genes included in each genomic interval from the Ensembl gene annotation 100 in Biomart (http://www.ensembl.org/biomart/martview/), and ii) assess their possible enrichment for any Gene Ontology (GO), Biological Processes (BP) and Human Phenotypes (HP) categories available in G-profiler83, retaining the categories that were significant at *P* < 0.05 after Benjamini–Hochberg corrections.

*Results.* A search on the CanFam3.1 dog genome assembly indicated that the outlier SNP TIGRP2P4604_rs8896093, identified when comparing Iberian and Dinaric wolves, mapped on chromosome 1, but had no protein-coding genes in its 100Kb flanking regions. However, when considering intervals of 1Mb, three protein-coding genes (Table S14) significantly enriched for GO categories (Table S15) linked to biological processes such as immune response and calcium homeostasis modulation were detected 0.69-0.76 Mb from the SNP (Table S16). The outlier SNP BICF2P1183096, identified when comparing Dinaric and Italian wolves, mapped on chromosome 8 (Table S3) in a 100-kb genomic region including six protein-coding genes (Table S14), of which three were significantly enriched for GO categories (Table S15) linked to biological processes such as cellular metabolism and stress response, kidney filtration ability and muscular regulation (Table S16). The outlier SNP BICF2P137558, identified when comparing Iberian and Italian wolves, mapped on chromosome 25 (Table S3) in a 100-kb genomic region including four other protein-coding genes (Table S14), three of which were significantly enriched for GO categories (Table S15) linked to biological processes such as high altitude adaptation, photo-reaction to nocturnal darkness, bone ossification, and immune response (Table S16).

*Discussion.* Despite the limited number of SNPs compared to genome-wide studies^4-5^ and the absence of strong environmental differences, we detected one locus under potential selection flanked by genes enriched for several GO categories for each pairwise comparison between the three wolf populations. Although the three populations share broadly similar Mediterranean environments, they may be experiencing different selective pressures at the local level, which have been described as the basis for the genetic distinction of different wolf ecotypes^2,6^. Earlier analyses of wolves across Europe have suggested parallel selection for similar traits acting on different genes in divergent populations^2,6^. Although various processes including genetic drift can confound signals of selection, the finding of separate genes coding for similar traits (e.g., immune function, kidney filtration) in our study could be further examined to evaluate possible patterns of selection. Rapidly advancing genomic methods have greatly increased the opportunities to investigate such patterns of selection, which have been reported in diverse species^7-8^. However, we cannot exclude the possibility that genetic drift or other factors have influenced the results, and future research on a larger data set, for example based on transcriptome analyses (e.g., Campbell-Staton *et al.*^7^) or whole genomes (e.g., Plassais *et al.*^9^), could provide further information.

**Supplemental Note S2. Estimates of wolf population divergence times**

*Methods.* We used SNP data to run Approximate Bayesian Computation simulations (ABC) implemented in the software Diyabc 2.1.0^10^. We modeled plausible demographic scenarios and estimated divergence times (in generations) among the three wolf populations, and for these analyses we excluded the three phenotypic loci, SNPs identified by BayeScan as being under possible selection, and all the individuals in each wolf population identified as admixed. Because the three southern European wolf populations appear to have diverged closely in time, and their effective sizes steadily declined in the last ten thousands of years^2,4,11-12^, eight demographic scenarios were examined (Fig. S2), assuming that the three populations (WIB, WIT and WDIN) split simultaneously (Scenarios 1, 2, 3 and 4) or sequentially (Scenarios 5, 6, 7, and 8) and that they experienced simultaneous (Scenarios 2 and 6), sequential (Scenarios 3, 4, 7 and 8) or no (Scenarios 1 and 5) bottlenecks. Altogether 6 x 10^6^ simulations were run for each scenario using uniform prior distributions of the effective population size and time parameters with default settings (Table S17). Summary statistics (proportions of zero values, mean of non-zero values, variance of non-zero values and mean of complete distributions) were estimated for genetic diversities and *F*_ST_ distances. Scenarios were compared by estimating posterior probabilities with the logistic regression method in Diyabc using 1% of the simulated datasets. For the best models, posterior distributions of the parameters were estimated with a logit-transformed linear regression on the 1% simulated datasets closest to the observed data. Scenario confidence was evaluated by comparing observed and simulated summary statistics. Finally, the goodness-of-fit of the posterior parameters for the best performing scenarios was tested via the model checking option with default settings, and significance was assessed after Bonferroni correction for multiple testing^10^.

*Results.* ABC simulations showed the highest support for scenario number 7 (sequential population splitting with subsequent bottlenecks), which performed better than the other seven models examined and showed *P*-values of the summary statistics that were not significantly different from simulations for all the posterior parameters after Bonferroni’s corrections (Table S18). Under scenario 7, the mean values of the divergence times suggested that separation among the three wolf populations occurred relatively close in time (Fig. S3 and Table S19). Our results indicated that Dinaric wolves have been isolated from the other two populations for about 2,670 generations (5% quantile (q050) = 320 − 95% quantile (q950) = 4,790), and Iberian and Italian wolves for the last 2,370 generations (5% quantile (q050) = 237 − 95% quantile (q950) = 4,690) (Fig. S3 and Table S14). Therefore, assuming a generation time of 3.0^13^ or 4.5^14^ years, respectively, our results suggest that Dinaric wolves started to diverge from the others *c.* 8,010 (960–14,370) or 12,015 (1,440–21,555) years ago, whereas Italian wolves started to diverge from Iberian wolves *c.* 7,110 (711–14,070) or 10,665 (1,067–21,105) years ago (Fig. S3 and Table S14). Under scenario 7, the current effective population sizes (N3, N2 and N1; Fig. S3) strongly declined, showing a reduction of *c*. 5.7 times in the Dinaric, *c*. 5.4 times in the Iberian and *c*. 6.3 times in the Italian wolf populations after their bottlenecks. These bottlenecks were estimated to have started *c.* 6,480–9,720 years ago for Dinaric wolves (from 513–13,980 to 770–20,970), *c.* 3,900-5,850 years ago for Iberian wolves (from 702–10,950 to 1,053–16,425), and *c.* 3,480-5,220 years ago for Italian wolves (from 303–4,470 to 455–6,705), depending on the generation time applied (3 *vs.* 4.5 years, respectively; Table S19).

*Discussion.* Several studies have been performed to estimate divergence times in European wolf populations based on analyses of different molecular markers such as mtDNA^15^, microsatellites^12^, SNPs^2^, or whole genomes^5^, which have produced different patterns and estimates. Our results suggest that the three wolf populations separated after the last glacial maximum, with the Dinaric population separating first (*c.* 8.000-12.000 years ago), followed by the Italian and Iberian populations (7.000-10.000). These results are consistent with those from the recent study of Silva *et al.*^5^ based on analyses of whole genomes from Croatian, Italian, and Iberian wolves. However, our findings and those of Silva *et al.*^5^ vary depending on the generation time applied, which was either 3 years^13^ or 4.5 years^14^. As expected, our results also had very wide confidence intervals, likely due to the small number of loci used for the analyses. Moreover, the bias toward loci with high *F*_ST_ values may also have affected the results. Nonetheless, our marker set was able to model bottleneck and genetic drift events that have affected these wolf populations, mainly in Italy and Iberia^2,16-18^.

**References**

1. Foll M, Gaggiotti O (2008). A genome-scan method to identify selected loci appropriate for both dominant and codominant markers: a Bayesian perspective. Genetics. 180: 977-993.

2. Pilot M, Greco C, vonHoldt BM, Jędrzejewska B, Randi E, Jędrzejewski W, Sidorovich VE, Ostrander EA, Wayne RK (2014). Genome-wide signatures of population bottlenecks and diversifying selection in European wolves. Heredity 112: 428-442.

3. Galaverni M, Caniglia R, Pagani L, Fabbri E, Boattini A, Randi E (2017). Disentangling Timing of Admixture, Patterns of Introgression, and Phenotypic Indicators in a Hybridizing Wolf Population. Mol. Biol. Evol. 34: 2324-39.

4. Gopalakrishnan S, *et al*. (2017). The wolf reference genome sequence (*Canis lupus lupus*) and its implications for *Canis spp*. population genomics. BMC Genomics 18:495.

5. Silva P, Galaverni M, Ortega-Del Vecchyo D, Fan Z, Caniglia R, Fabbri E, Randi E, Wayne R, Godinho R (2020). Genomic evidence for the Old divergence of Southern European wolf populations. Proc. R. Soc. B 287: 20201206.

6. Stronen AV, *et al*. (2015). Genome-wide analyses suggest parallel selection for universal traits may eclipse local environmental selection in a highly mobile carnivore. Ecol. Evol. 5: 4410-4425.

7. Campbell-Staton SC, Winchell KM, Rochette NC, Fredette J, Maayan I, Schweizer RM, Catchen J. 2020. Parallel selection on thermal physiology facilitates repeated adaptation of city lizards to urban heat islands. Nat. Ecol. Evol. 4: 652-658.

8. Taylor RS, Manseau M, Horn RL, Keobouasone S, Golding GB, Wilson PJ. 2020. The role of introgression and ecotypic parallelism in delineating intraspecific conservation units. Mol. Ecol. 29: 2793-2809.

9. Plassais J, Kim J, Davis BW et al. (2019). Whole genome sequencing of canids reveals genomic regions under selection and variants influencing morphology. Nat. Commun. 10: 1489.

10. Cornuet J-M, *et al*. (2014). Diyabc v. 2.0: a software to make approximate Bayesian computation inferences about population history using single nucleotide polymorphism, DNA sequence and microsatellite data. Bioinformatics 30: 1187-1189.

11. Fan Z, *et al*. (2016). Worldwide patterns of genomic variation and admixture in gray wolves. Genome Res. 26: 1-11.

12. Montana L, Caniglia R, Galaverni M, Fabbri E, Ahmed A, Bolfíková BČ, Czarnomska CD, Galov A, Godinho R, Hindrikson M, Hulva P, Jędrzejewska B, Jelenčič M, Kutal M, Saarma U, Skrbinšek T, Randi E (2017). Combining phylogenetic and demographic inferences to assess the origin of the genetic diversity in an isolated wolf population. PLoS ONE 12: e0176560.

13. Skoglund P, Gotherstrom A, Jakobsson M (2011). Estimation of population divergence times from non-overlapping genomic sequences: examples from dogs and wolves. Mol. Biol. Evol. 28: 1505-1517.

14. Mech LD, Barber-Meyer SM, Erb J (2016). Wolf (Canis lupus) generation time and proportion of current breeding females by age. PLoS ONE 11: e0156682.

15. Pilot M, *et al*. (2010). Phylogeographic history of grey wolves in Europe. BMC Evol. Biol. 21: 10-104.

16. Lucchini V, Galov A, Randi E (2004) Evidence of genetic distinction and long-term population decline in wolves (*Canis lupus*) in the Italian Apennines. Mol. Ecol. 13: 898-902.

17. vonHoldt BM, Pollinger JP, Earl DA, Knowles JC, Boyko AR, Parker H, Geffen E, Pilot M, Jedrzejewski W, Jedrzejewska B, Sidorovich V, Greco C, Randi E, Musiani M, Kays R, Bustamante CD, Ostrander EA, Novembre J, Wayne RK (2011). A genome-wide perspective on the evolutionary history of enigmatic wolf-like canids. Genome Res. 21:1294-305.

18. Stronen AV, *et al*. (2013). North-south differentiation and a region of high diversity in European wolves. *PLoS ONE*, 8(10), e76454.

19. Peakall, R. & Smouse, P. E. GenAlEx 6.5: genetic analysis in Excel. Population genetic software for teaching and research-an update. *Bioinformatics* **28**, 2537-2539 (2012).

20. Pritchard, J. K., Stephens, M. & Donnelly, P. Inference of population structure using multilocus genotype data. *Genetics* **155**, 945-959 (2000).

21. Alexander, D. H., Novembre, J. & Lange, K. Fast model-based estimation of ancestry in unrelated individuals. *Genome Res.* **19,** 1655-1664 (2009).

22. Jakobsson, M. & Rosenberg, N. A. Clumpp: a cluster matching and permutation program for dealing with label switching and multimodality in analysis of population structure. *Bioinformatics* **23**, 1801-1806 (2007).

1. Rosenberg, N. A. Distruct: A program for the graphical display of population structure. *Mol. Ecol. Notes* **4**, 137-138 (2004).
2. Jombart, T. Adegenet: a R package for the multivariate analysis of genetic markers. *Bioinformatics* **24**, 1403-1405 (2008).

***Supplemental Figure legends***

**Figure S1.** Structure and Admixture results. **(a)** Bar plots for individual *q*_i_-values obtained running Structure 2.3.4^20^ with the 98,004 SNP-genotypes of the parental populations (J: jackals; D: dogs; WDIN: Dinaric; WIB: Iberian and WIT: Italian wolves), assuming *K* = 5 clusters and the “*Admixture*” and “*Independent allele frequencies*” models. Bar plots were obtained concatenating data from the five independent runs using Clumpp 1.1.1^22^ and graphically displayed using Distruct 1.1^23^. **(b)** Bar plots for individual *q*_i_-values obtained running Admixture 1.23^21^ with the 98,004-SNP genotypes of the parental populations (J: jackals; D: dogs; WDIN: Dinaric; WIB: Iberian and WIT: Italian wolves), with default parameter settings and assuming *K* = 5 clusters. **(c)** Cross validation errors for *K* from 1 to 10 obtained running Admixture 1.23^21^ with the 192-SNP genotypes of the five reference populations (J: jackals; D: dogs; WDIN: Dinaric; WIB: Iberian and WIT: Italian wolves). **(d)** Bar plots for individual *q*_i_-values obtained running Admixture 1.23^21^ with the 192-SNP genotypes of the parental populations (J: jackals; D: dogs; WDIN: Dinaric; WIB: Iberian and WIT: Italian wolves), with default parameter settings and assuming *K* = 5 clusters. **(e)** Bar plots for individual *q*_i_-values obtained with Admixture 1.23^21^ for the 192-SNP genotypes of the five reference populations and 10 first-generation (F1) hybrid and first-generation backcross (BC1) genotypes simulated for each pairwise combination of canid groups, with default parameter settings and assuming *K* = 5.

**Figure S2.** Graphical representation of the eight simulated demographic scenarios run in Diyabc 2.1.0^10^. Scenarios 1,2, 3 and 4 assumed that the three wolf populations split simultaneously, scenarios 5, 6, 7, and 8 that they diverged sequentially, and that populations experienced simultaneous (Scenarios 2 and 6), sequential (Scenarios 3, 4, 7 and 8) or no (Scenarios 1 and 5) bottlenecks.

**Figure S3.** Graphical representation of the best performing demographic scenario (number 7) simulated in Diyabc 2.1.0^10^. This scenario assumed sequential population splitting with sequential bottlenecks. The resulting population sizes and divergence times were reported using a generation time of g = 3.0 or g = 4.5 years. N1-N2-N3: Italian-Iberian-Dinaric wolf post-bottleneck effective population sizes; N1b-N2b-N3b: Italian-Iberian-Dinaric wolf pre-bottleneck effective population sizes; NA effective population size of the initial population; t1: the divergence time from the common ancestor in thousands of generations; t2: the divergence time between the Italian and Iberian wolf populations in thousands of generations; db3: bottleneck duration for the Dinaric wolf population; db2: bottleneck duration for the Iberian wolf population; db1: bottleneck duration for the Italian wolf population.

**Figure S4.** Scores of individual 178-SNP invasive and non-invasive genotypes of wild and domestic canids plotted on the first two axes of a Principal Coordinates Analysis (PCoA) computed using GenAlex 6.502^19^. The first axis, which explained more than 35% of the total genetic variability, clearly separated Italian wolves (WIT; left side) from all the other canids (right side), whereas the second axis, which explained more than 20% of the total genetic variability, clearly separated dogs (bottom right side), from fox, jackal, Dinaric (WDIN) and Iberian (WIB) wolf (bottom right side) samples. Notably, non-invasive genotypes clustered with profiles from their respective reference groups (grey dots), and admixed individuals (Hy-IT, black dots) were positioned between parental groups, with older-generation backcrosses located closer to wild parental groups.

**Figure S5.** Structure and Adegenet results obtained with the 178 SNPs that performed successfully in the Fluidigm test. **(a)** Bar plots for the 158 individual *q*_i_-values obtained through assignment with the 178-SNP genotypes. Each individual is represented by a vertical line partitioned into coloured segments, whose length is proportional to the individual coefficients of membership (*q*_i_) in the jackal, dog and wolf clusters inferred by Bayesian assignment analyses performed in Structure 2.3.4^20^, assuming *K* = 5, no prior information (option *Usepopinfo* not activated) and choosing the “*Admixture*” and “*Independent Allele Frequency*” models. J: jackals; D: dogs; WDIN: Dinaric wolves, WIB: Iberian wolves; WIT: Italian wolves. Bar plots were obtained concatenating data from the five independent runs using Clumpp 1.1.1^22^ and graphically displayed using Distruct 1.1^23^. **(b)** Bar plots for the 158 individual *q*_i_-values obtained through Structure 2.3.4^20^ assignment for the canid reference populations with the 178-SNP genotypes, assuming *K* = 5 clusters: jackals (J), dogs (D), Dinaric wolves (WDIN), Iberian wolves (WIB), Italian wolves (WIT), and 10 first-generation (F1) hybrid and first-generation backcross (BC1) genotypes simulated for each pairwise combination of canid groups. Bar plots were obtained concatenating data from the five independent runs using Clumpp 1.1.1^22^ and graphically displayed using Distruct 1.1^23^. Results from **(c)** a multivariate discriminant analysis (PC performed with the “*dudi.pca*” function) in the Adegenet 2.3.4^24^ package and from **(d)** a Bayesian assignment approach in Structure 2.3.4^20^ (assuming *K* = 5 and the “*Admixture*” and “*Independent allele frequencies*” models), obtained with 178-SNP genotypes for the five reference populations (J: jackals; D: dogs; WDIN: Dinaric; WIB: Iberian; WIT: Italian wolves) together with 33 additional canid genotypes categorized as non-admixed (W, grey dots) or admixed (Hy, black dots) in earlier studies (n = 10 Italian and n = 9 Dinaric wolves, n = 4 Italian wolf *x* dog (HyIT), n = 4 Dinaric wolf *x* dog (HyDIN), n = 4 Iberian wolf *x* dog (HyIB), and n = 2 jackal *x* dog individuals (HyJ-D)). Bar plots were obtained concatenating data from the five independent runs using Clumpp 1.1.1^22^ and graphically displayed using Distruct 1.1^23^.
